# Preserving edible biodiversity through indigenous dairy emulsions: valorization of traditional milk systems for a resilient future

**DOI:** 10.3389/fnut.2025.1693995

**Published:** 2025-10-23

**Authors:** Anand Kumar, Muskan Yadav, Sadaqat Ali, Itu Dutta, Selva Kumar T, Vidhu Yadav, Tharindu Trishan Dapana Durage, Fahad Saad Alhodieb, Saleh A. Alsanie

**Affiliations:** ^1^College of Food Science and Technology, Guangdong Ocean University, Zhanjiang, China; ^2^Department of Food and Nutrition and Food Technology, Institute of Home Economics, University of Delhi, New Delhi, India; ^3^Vel Tech Rangarajan Dr. Sagunthala R&D Institute of Science and Technology, Chennai, India; ^4^School of Nutrition and Food Sciences, Louisiana State University Agricultural Center, Baton Rouge, LA, United States; ^5^Department of Basic Health Sciences, College of Applied Medical Sciences, Qassim University, Buraydah, Saudi Arabia

**Keywords:** indigenous dairy systems, emulsion, valorization, functional foods, nanoemulsions, sustainable food systems, traditional dairy emulsions

## Abstract

Traditional dairy emulsions such as ghee, laban, reyab, and fermented camel milk are produced using locally adapted livestock breeds and unique microbial consortia, reflecting centuries of ecological adaptation. These products are increasingly positioned as functional foods that integrate traditional practices with modern nutritional needs, thereby enhancing dietary diversity and sustainability. These systems are structurally complex and nutritionally dense, containing bioactive compounds, natural emulsifiers, and probiotics. They contribute to regional food resilience and edible biodiversity through bioactive compounds, natural emulsifiers, and probiotics. This paper explores the dynamic role of these emulsions in dairy food systems, their biochemical makeup, socio-cultural relevance, and technological avenues for their valorization. Advances in nano and micro emulsion methods, ultrafiltration, and spray drying are investigated for their potential to improve bioavailability, stability, and application while maintaining traditional values. Recent research has shown that when used correctly, these technologies can preserve or even enhance their health-promoting properties, allowing them to be incorporated into modern diets and treatment formulations. However, their value must be based on ethical considerations for local producers, the preservation of microbiological and cultural variety, and a governmental framework that encourages small-scale, decentralised innovations. When treated with cultural sensitivity and scientific precision, these technological interventions for the valorisation of traditional dairy systems can increase their nutritional and functional value while conserving their essential identity. These emulsions have the potential to bridge the gap between tradition and innovation, facilitating for the development of diversified, robust, and culturally grounded nutritional practices.

## 1 Introduction

While food security is influenced by multiple factors, biodiversity plays a crucial role in ensuring dietary variety and preventing food insecurity, especially in regions facing climatic challenges. Edible biodiversity is essential for maintaining diverse diets and preventing malnutrition during periods of agricultural scarcity. It is vital to preserve local food systems, such as traditional dairy emulsions, which provide vital nutrition, particularly in areas where conventional agriculture struggles. These systems, supported by indigenous livestock, play a critical role in food security by diversifying dietary sources and ensuring resilience (1, 2). The globalized food industry has seen an upward trend in consumption of ultra-processed foods that rely on high-yield, high-energy raw materials and the production of which exacerbates agrobiodiversity destruction and reduces dietary diversity (3). From contemporary research we can concur that there is a positive correlation between dietary diversity and quality of diets. The positive effect of consuming a diverse diet is not limited to immediate health outcomes but also ensures lessening of burden from one stream of food processing and production, thereby reducing ecological strain (4).

Given that the population is projected to reach 8.3 billion by 2030, edible biodiversity becomes even more crucial. With such an increment, it is important that populations have access to adequate nutrition at all time periods. Production and supply systems must be optimized to prevent food insecurity, in this animal derived products such as milk are a vital pillar holding up food security in many regions of the world (5). Current forecast of trends in dairy industry are indicative of an interdisciplinary shift in approach to include automation, greater attention to quality standards, intensive production, and cost reduction by confining animal production systems. The expansion of the dairy industry in the global south is set out to revolve around mechanized and standardized production streams that are optimized to enhance product quality (6). The rush to expand production in dairy industry is often accompanied by increase in CO_2_ emissions, imbalance of trade, decline in animal welfare, high input costs, and upheaval of rural and cultural landscapes. There is a challenge to efficiently manage resources while upholding milk quality in a sustainable manner. The current approach to expansion is detrimental not only to the environment but also to the people involved, with a significant increase in labour, feed and infrastructure requirement. Therefore, there is an urgent need to explore for alternatives that can meet maximum needs with minimum damage. Industrial dairy systems contribute significantly to environmental burdens, with estimates suggesting that up to 4% of global greenhouse gas emissions originate from this sector. Moreover, the reliance on intensive feed systems has been shown to reduce livestock genetic diversity, raising concerns about long-term resilience. These figures underscore the urgency of conserving indigenous systems that sustain both biodiversity and ecological balance (7, 8).

**Figure 1:**
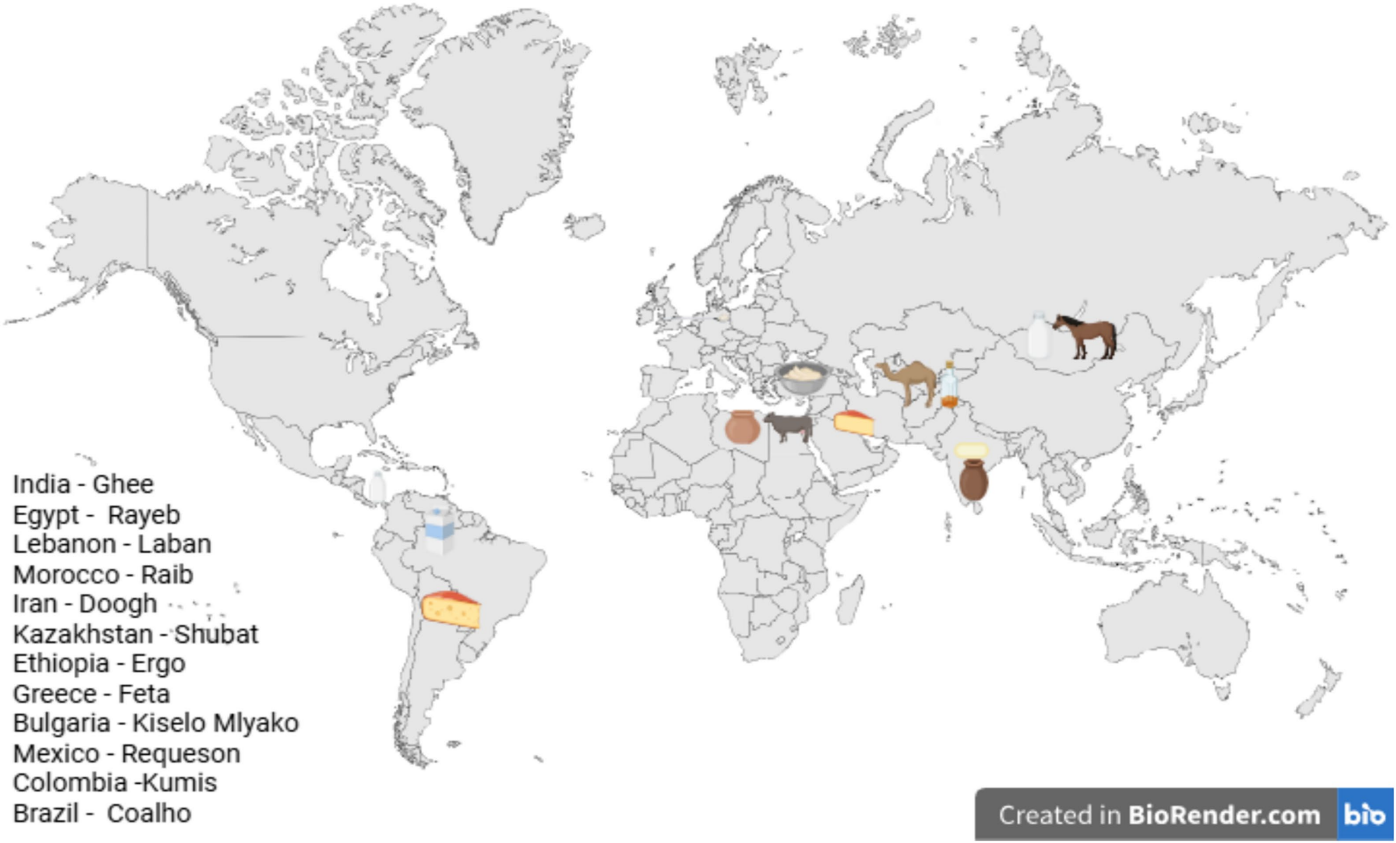
Diversity of traditional dairy emulsions across globe.

A product arising from local customs, adapted to specific resident livestock and local ecosystem are indigenous dairy emulsions. These are culturally relevant products produced by traditional techniques and hold up well against globalized trends. These unique products are produced through the emulsification of milk fats and fermentation techniques. Contemporary focus can be shifted to indigenous dairy emulsions that go beyond being mere means of sustenance. Dairy emulsions embody the generational know-how which not only are pro-diversity approach to dietary diversity but also lessen pressure on traditional food production systems. With appropriate policy support, sustainable commercial upscaling, and stringent microbiological and optimization studies we may bring these traditional emulsions to the forefront. Policy interventions such as Geographical Indication (GI) certification, targeted subsidies for small-scale dairy farmers, and the inclusion of indigenous emulsions in national dietary guidelines provide an enabling framework for sustainable commercialization. Such measures ensure that traditional producers benefit economically while safeguarding cultural heritage. In this review thus, an attempt has been made to highlight the inherent richness of dairy emulsion systems, focusing on contextualizing dairy emulsions by region specific case studies, expanding on their cultural and nutritional significance, and in due course their functional properties, challenges in production and future outcomes have been highlighted (Figure 1).

## 2 Overview of indigenous dairy emulsions

### 2.1 Definition and classification of dairy emulsions

Dairy formulations are multiphase complexes where emulsifying agents, usually milk proteins, stabilize immiscible fluids, usually water and oil, in a diffused structure. Emulsions are often classified as either oil-in-water (O/W) or water-in-oil (W/O) varieties according to their scattered and uninterrupted phases, respectively. Most dairy-based emulsions are classified as oil-in-water, meaning that globules of milk fat are dispersed in an aqueous matrix. Phospholipid membranes and proteins like casein and whey act as stabilizers for these colloidal preparations (9, 10).

Milk is an emulsion of fat globules in aqueous medium, stabilized by Proteins of milk – casein and whey- tend to form viscoelastic films at the interface of immiscible liquids such as oil and water, acting as surfactants reducing surface tension, and thereby preventing droplet coalescence or flocculation. Manipulation of protein conformation can be carried out to enhance their emulsification properties (11–14).

The dispersed phase, the dispersion medium and the emulsifier at the interface that stabilises the system are the three phases that make up food emulsions. The dispersed phase acts as a carrier for lipid-soluble substances such as antioxidants and flavour compound while the dispersion medium plays a crucial role as a solvent for molecules responsible for provision of mineral content, vitamins, carbohydrates, and acids (15).

### 2.2 Cultural and nutritional relevance

The dairy sector is a goldmine of value-addition, offering diverse product platforms including fermented milk beverages, yogurt, ghee, butter, cheese, and whey-derived emulsions (10). These products are known to supply essential amino acids, micronutrients and calcium that play a vital role in the nutrition of all age groups (8).

Due to their nutritional benefits, indigenous fermentates such as yogurt, laban, tarag, and koumiss occupy an important space. They are abundant in bioactive substances including myo-inositol, alpha-hydroxyisocaproic acid, and gamma-aminobutyric acid (GABA), which have demonstrated a number of physiological advantages, such as immune-modulation and lowering of blood pressure the microflora in the GI tract can be modulated thereby improving intestinal health by consumption of these efficient probiotic and prebiotic delivery vehicles. Some identified probiotics in these emulsions are *Streptococcus thermophilus*, *Lactobacillus kefiranofaciens*, *E. lactis*, and *Enterococcus durans,* helping to balance the immune system thereby providing early advantages in diseases such as Crohn’s disease (16).

Beyond nutritional and functional attributes, consumer perception plays a decisive role in valorization. Sensory studies show, for example, that camel milk flavored with cinnamon and date pulp achieved higher acceptability among consumers, while laban fortified with coriander oil retained desirable texture and improved flavor perception. These examples highlight the need to integrate sensory quality with nutritional enhancement in future valorization strategies. Bioactive compounds are added to dairy emulsions in a bid to fortify them by techniques such as, liposomal confinement and nanoencapsulation. The focus is on maintaining sensory qualities while increasing the physiological availability, prolonging the shelf life and ensuring controlled release of compounds (17).

### 2.3 Role in traditional food systems and heritage preservation

From Middle Eastern villages to the Mongolian steppes, classic dairy formulations are ingrained in the eating habits of many tribes, serving as both dietary supplements and emblems of culture. Such items rely on a naturally occurring fermentation by local rather than commercial starting cultures and are frequently the result of ecologically responsive processes, such as maturation in leather bags, earthenware, or wooden containers (18). Examples like koumiss, airag, and tarag combine yeasts and lactic acid bacteria to create moderately alcoholic emulsions with unique sensory characteristics and a long shelf life.

Numerous traditional culinary traditions are likewise based on the safe keeping of milk in colloidal form. In the past, methods including salting, air drying, and cold storage—which uses an acidic pH to prevent spoiling—have been used to guarantee the safety and durability of milk when refrigeration is not available. More specifically, fermentation turns fresh milk into emulsions that are stable, such as cheese, yoghurt, and laban, which improves flavour, digestibility, and functional value while halting degradation (19).

Concern regarding the loss of microbiological and functional validity as a result of industrial standardization is growing, especially by incorporation into international food supply chains. The legacy of such products is threatened by the loss of native starter cultures, changes in milk composition, and departure from traditional practices. It is now crucial to preserve these emulsions using biobanking, DNA fingerprinting, and microbiome analysis to protect heritage dairy products from identity loss and commercial adulteration (13).

Traditional dairy emulsions can be broadly classified as fermented milk emulsions (e.g., laban, rayeb), clarified fat-based emulsions (e.g., ghee), curd/cheese emulsions with whey components (e.g., halloumi) and animal-specific fermented milks (e.g., shubat and gariss). These product categories do not have strict demarcations and may feature similarities or differ from region to region; the properties of these products mainly vary based on geography, climate, livestock breed and local customs. Some of them have been discussed in the following section.

## 3 Case studies of region-specific dairy emulsions

### 3.1 Fermented camel milk (e.g., Gariss, Shubat)

Camel milk differs significantly from the milk of other ruminants, offering lower levels of cholesterol and sugar, while providing higher amounts of minerals such as sodium, potassium, iron, copper, zinc, and magnesium, as well as elevated vitamin A, B2, C and E content. Its distinctive composition and reported health benefits set it apart. The milk itself is opaque white, has a normal odor, and a salty taste—the white color results from the fine homogenization of fat particles throughout the milk (20).

Camel colostrum, rich in lactoferrin and free of β-lactoglobulin, adds value to fermented dairy products. Fermented samples containing colostrum exhibit low-molecular-weight peptides, especially immunoglobulins (IgG, IgM, IgA), which improve both texture and flavour (21). Camel milk and its derivatives exhibit a range of bioactivities, including antimicrobial, antioxidant, anti-obesity, anti-hypertensive (ACE-inhibitory), antidiabetic, anticancer, and anti-inflammatory effects (22–24). Additionally, it has been shown to help lower elevated levels of bilirubin, globulin, and granulocytes (20). These products can regulate lipid metabolism, improve gut microbiota, and enhance liver antioxidant function (19, 25–27).

In arid regions, camel milk plays a vital nutritional role, delivering exceptional health benefits. It is especially noted for its high levels of protective proteins, including lactoferrin, lactoperoxidase, immunoglobulins, and lysozyme. However, transforming camel milk into dairy products remains challenging due to its low *κ*-casein, large casein micelles, and absence of β-lactoglobulin, making the production of cheese and yogurt technically difficult. Specialized processing technologies and further research are needed to fully utilize camel milk’s potential (28).

Camel milk fermentation leads to the formation of free amino acids, bioactive peptides, and lactoyl/acetylated amino acids. *L. helveticus*, in particular, generated peptides rich in proline, with ACE-inhibitory activity, which may help manage hypertension (29). Flavouring fermented camel milk with cinnamon, doum, or red quinoa flour has been shown to increase total phenolic content, antioxidant activity, and consumer acceptability (20, 30). Ajwa date pulp, when used in freeze-dried fermented camel milk, also improved polyphenolic activity, mineral levels, and ACE-1 inhibition (16). Fermented camel milk is known for enhancing digestive health, cholesterol regulation, and immune function (31). Lactic acid bacteria (LAB), central to camel milk fermentation, are recognized for their antioxidant, hypotensive, antidiabetic, and anticancer capabilities, and for improving lactose digestion (11, 32).

Fermented camel milk is traditionally prepared in various countries under local names such as Gariss (Sudan), Shubat (Kazakhstan), Dhanaan (Ethiopia), and Chal (Iran) (12, 17, 33, 34). Gariss is traditionally produced without heat treatment or added starter cultures. In Sudan and Somalia, it is known as *Gariss*, *hameedh*, or *humadah*, all meaning “sour.” This naturally fermented milk often contains ethanol because of acid-alcohol fermentation. It is fermented in large skin bags (called *Siin*), which retain some of the previously soured milk to initiate fermentation. If a starter is unavailable, a few black cumin seeds (*Nigella sativa*) and an onion bulb are added (34).

Gariss typically contains 2.8–5% fat, 10–11% total solids, pH between 3.6 and 5.9, and lactic acid levels of 2.2–2.3%. It is produced through semi-continuous or fed-batch fermentation in *Siin* bags. Acetaldehyde, a key flavor compound, increases slightly during the first 10 days of storage before declining. The presence of lactic, acetic, and citric acids contributes to its antimicrobial properties, while volatile fatty acids and acetaldehyde enhance its pleasant flavour (35, 36).

Shubat is a traditional fermented camel milk drink with roots in Kazakhstan, culturally significant throughout Central Asia (37). Thanks to camel milk’s unique composition—rich in unsaturated fatty acids, bioactive proteins, and vitamins—Shubat provides an excellent environment for microbial activity. Metagenomic analysis of Shubat reveals a diverse microbial ecosystem, dominated by Lactobacillus (16.22–82.76%) and Lactococcus (4.45–50.43%). Key species include *Lactobacillus helveticus*, *Lactococcus lactis*, and *Leuconostoc mesenteroides*, along with others like *Lb. kefiranofaciens* and *Lc. Raffinolactis* (11, 21, 25, 31, 32, 38). Among fungi, *Geotrichum candidum* is predominant, followed by *Brettanomyces bruxellensis* (39). LAB play a major role in acid production, flavor development, and overall product quality (40).

Dhanaan is camel milk fermentate from Somali region of Ethiopia, produced especially when milk is abundant or travel is planned, due to its extended shelf life. Fresh camel milk is poured into a clean, smoked plastic container (called a *Jerry can*) and left to ferment naturally for 48–72 h at 25–35 °C. The containers are smoked for 20–30 min using embers of *Olea afana* wood, imparting a distinct smoky flavor. However, inconsistent preparation practices often result in variable quality and safety (12).

Chal, produced in Golestan Province, Iran, is another spontaneously fermentate produced from camel milk with higher antioxidant activity than bovine milk. Fermentation with *Leuconostoc lactis* significantly boosts DPPH and ABTS radical scavenging capacity. Among strains tested for its production, *Leuconostoc lactis SM10* achieved the highest sensory acceptance. Nine catalase-negative bacteria have been isolated from Chal, including *Lactobacillus plantarum*, *L. kefiri*, *L. gasseri*, *Leuconostoc lactis*, *Weissella cibaria*, and *Enterococcus faecium*. Predominant lactic acid bacteria strains are *L. helveticus*, *L. acidophilus*, and *L. paracasei*. Their presence has been studied, results indicate that their numbers reached over 7.0 log CFU/mL within 16 h, exceeding 8.5 log CFU/mL by 104 h (22, 33, 35).

Efforts have also been made to incorporate camel milk into processed cheese products. One strategy involves ultrafiltration of whole camel milk to obtain a concentrated retentate, which is then fermented with yogurt cultures and rennet to produce a camel cheese base, which when processed to a spread has been found to have acceptable sensory attributes, and appropriate firmness (37).

### 3.2 Ghee and its variants (traditional clarified butter systems)

Ghee, a form of clarified milk fat, is a staple across South Asia and parts of Central Asia, popular for its rich aroma, nutty taste, and nutritional value. Traditionally prepared through heat clarification of milk fat, ghee can be made from the milk of cows, buffaloes, goats, and yaks (41). Ghee contains a range of fatty acids, including short-chain (SCFAs), medium- and long-chain (MCFAs and LCFAs), and very-long-chain fatty acids (VLCFAs). Notable examples include butyric acid (BA) as the predominant SCFA, palmitic acid (PA) as the main saturated fat, and oleic acid (OA) as the major unsaturated fat. These fatty acids contribute to important physiological functions: PA and OA help regulate blood lipid levels and may reduce hypercholesterolemia risk; linoleic acid (LA) and *α*-linolenic acid (LNA) provide anti-inflammatory effects; and CLA, derived from LA, is linked to atherosclerosis prevention and developmental health support. The health implications of ghee remain contested. Traditional beliefs emphasize its nutritive and medicinal properties, yet earlier dietary guidelines associated ghee with cardiovascular risk due to its saturated fat content. However, recent meta-analyses indicate no consistent link between high-fat dairy intake and elevated LDL cholesterol. This evolving evidence highlights the complexity of evaluating ghee’s health effects (13, 42, 43).

Among different types of ghee, yak ghee is especially important in Tibetan cuisine, accounting for 15–32% of the daily fat intake among herding communities. It delivers key fat-soluble vitamins—A, D, E, and K—alongside functional fatty acids such as arachidonic acid and conjugated linoleic acid (CLA), both essential for brain development, neural function, and overall growth in children. It is a potent source of bioactive lipids, reportedly containing up to eight times more than regular butter. Studies indicate that as yak ghee ages, concentrations of certain functional lipids increase, enhancing its nutritional profile. In one study, 64 lipid markers were identified in Zhongdian yak ghee, highlighting its complex lipid composition and health potential (41).

Ghee’s signature flavor and aroma result from various volatile compounds such as alcohols, aldehydes, acids, ketones, lactones, and carbonyls formed during the heating process. Lactones and carbonyls especially responsible for the caramelized, roasted notes. Preparation method, temperature, time, and storage conditions all affect this flavor profile. Using ripened ingredients like fermented cream or dahi intensifies microbial activity, enriching the taste. GC–MS analyses confirm that free fatty acids, esters, and other volatile compounds contribute to ghee’s distinctive sensory profile (44).

Despite their similarities, Tibetan butter and Indian ghee differ in processing and characteristics. Tibetan butter is processed more like traditional butter, while Indian ghee undergoes an extra clarification stage to eliminate water, making it a nearly anhydrous fat. In mechanized Indian ghee production, butter is first melted at 60 °C, followed by water removal at 90 °C, and final concentration at 110 °C (38). Tibetan butter production involves fermentation, maturation, churning, washing, and pressing, steps that help ensure low moisture and longer shelf stability. Adulteration is a shared concern for both products. Indian ghee is often diluted with vegetable oils or animal fats, whereas Tibetan butter may be adulterated with non-fat substances such as mashed potatoes (45).

During the production of ghee, a by-product known as ghee residue (GR) is formed, representing 10% of total yield. This residue, composed mainly of milk solids, is rich in protein, fat, and minerals. It also contributes to the overall antioxidant profile and flavor complexity of ghee due to thermal reactions during cooking; however, its dark color and intense taste have limited its direct applications. Safety risks in laban and rayeb, such as coliform and yeast contamination, can be addressed through pasteurization prior to fermentation, application of hazard analysis and critical control points (HACCP), and regulatory exemptions designed for artisanal producers. These measures balance food safety with the preservation of cultural authenticity (46).

The health benefits of ghee are closely linked to its molecular constituents. Butyric acid is recognized for its anti-inflammatory properties; pentadecanoic acid, an odd-chain fatty acid, supports membrane stability; and CLA has shown cardioprotective potential. Cutting-edge methods like untargeted lipidomics and metabolomics are being used to explore how processing affects ghee’s composition and health benefits (47). While ghee has traditionally been viewed with caution due to its high saturated fat content and presumed link to elevated LDL cholesterol, recent studies challenge this perspective. Evidence suggests that high-fat dairy consumption—including ghee—does not significantly increase LDL levels. In fact, its unique composition of functional fatty acids and bioactives may counterbalance the effects of saturated fats, positioning ghee as a functional fat rather than a health risk (46).

### 3.3 Laban and Rayeb (middle eastern fermented Milk emulsions)

Laban and Rayeb are traditional fermented dairy products widely enjoyed across North Africa and the Middle East. Laban is generally produced through the spontaneous fermentation of cow’s or goat’s milk, resulting in a smooth, tangy emulsion with a curd-like texture. In contrast, Rayeb—also known as *Laban Matared*—is a gravity-set sour milk in which natural coagulation occurs without the addition of rennet or mechanical stirring. Rayeb often serves as a precursor in the production of butter and cheese, whereas laban is typically consumed as a drink or incorporated into various traditional recipes (48, 49).

A regional variation known as Laban Zeer, originating from Upper Egypt (particularly Minia and Assuit), is a concentrated form of fermented milk. It is produced by pouring sour buttermilk into a porous clay jar (*zeer*), allowing excess liquid to drain and the mixture to thicken. This dense product is either eaten directly or used in dishes such as Kishk, a fermented blend of dairy and cereal. On average, Laban Zeer contains 25.98% total solids, 4.30% fat, 12.81% protein, 4.25% ash, and 4.58% salt. It is particularly rich in essential minerals like calcium, magnesium, zinc, and selenium, along with significant amounts of manganese, copper, and iron—although sodium levels are notably elevated. Amino acid profiling indicates high concentrations of essential amino acids, apart from methionine (38). Physicochemical analysis of traditional laban reveals a titratable acidity of 0.73%, pH around 4.16, total solids at 8.12%, and fat content of approximately 1.54%. The microbial profile is dominated by beneficial bacteria such as *Lactococcus*, *Streptococcus*, and both mesophilic and thermophilic *Lactobacillus* strains. However, coliforms, yeasts, and molds are frequently present, particularly in raw, non-pasteurized versions (50).

Traditional laban is often made by fermenting raw cow’s milk through naturally occurring microflora. In Tunisia and Libya, milk is placed in a goat-skin bag (*checoua* or *shakwa*), occasionally seeded with residues from prior batches to serve as a starter culture. The bag is then vigorously agitated to churn the contents, separating fat from the sour milk (49, 50). In rural Egypt, Laban Rayeb is prepared by allowing milk to ferment in shallow clay vessels (*mattered*) stored in dark rooms. Fermentation takes 12–24 h in warmer weather and longer in cooler conditions. This process results in spontaneous coagulation and the formation of a cream layer, after which the product can be consumed directly or used to make Karish cheese (48).

Rayeb shares many characteristics with laban but typically exhibits lower pH and lactose levels, resulting in a more acidic taste. It contains aromatic volatile compounds such as ethanol, acetaldehyde, diacetyl, and acetoin, which contribute to its distinctive flavor and aroma profile (51). Given Libya’s hot climate and limited access to refrigeration, traditional fermentation remains essential. Milk is typically stored at temperatures between 20 °C and 30 °C—ideal for lactic acid bacteria to thrive. In the production of Bio-Rayeb, a goat milk-based version, the addition of ingredients like coriander oil has been shown to enhance flavor and antioxidant capacity without affecting texture or acidity (52).

Both laban and rayeb offer notable functional health benefits as fermentation boosts their antioxidant activity by increasing levels of polyphenols and flavonoids. For example, Rayeb fortified with essential oils has demonstrated increased DPPH radical scavenging, indicating stronger antioxidant performance (32, 52). These fermented milk emulsions are also easier to digest, making them suitable for people with lactose intolerance or sensitivities to cow’s milk proteins. The use of goat milk, with its lower lactose and different protein structures, enhances gastrointestinal tolerance, and provides immune support (9, 43, 52). During fermentation, various intermediate compounds such as natural antibiotics, anticarcinogenic agents, and cholesterol-lowering substances are formed. Regular consumption is thus, tied to improved wellness of digestive system and reduced blood cholesterol, associated with presence of bioactive peptides (48, 49). Additionally, the stability of these products, arising from presence of organic acids such as lactic and acetic acid, is vital for shelf-life maintenance in hot regions with unreliable refrigeration. Organic acids prevent growth of spoilage microorganisms (32, 49).

### 3.4 Halloumi and whey by-products

Halloumi, also known by its Greek name *Χαλλούμι* or the Turkish *Hellim*, is a traditional semi-hard, unripened cheese originating from Cyprus. Historically produced using sheep’s and/or goat’s milk, cow’s milk has become more commonly incorporated in response to rising production needs (53). Since March 2021, Halloumi has held Protected Designation of Origin (PDO) status within the European Union, reinforcing its cultural and geographic authenticity. It has long been a dietary staple in Cyprus and is now widely recognized and exported internationally (13). The earliest documented mention of Halloumi appears in 1554 by Florio Bustron under the term “calumi,” with further reference made by the Kiprianos Church in 1778 (54).

Traditionally, Halloumi is distinguished by its semi-hard to hard, elastic texture, absence of a rind, compact and hole-free structure. Its color ranges from white to yellowish depending on the milk source—goat and sheep milk yield a whiter product, while cow’s milk contributes a more yellow tone (27). The cheese typically has a pH between 4.94 and 6.87 and contains 1 to 7% salt, averaging around 3%, which helps inhibit spoilage by limiting microbial growth (54). Halloumi may be eaten fresh, pan-fried, or preserved for extended periods in brined whey with 11–12% NaCl, which enhances its firmness and flavor profile (53). The cheese’s chemical makeup and nutritional value vary significantly based on the milk species used and the animals’ feeding regimen. Halloumi made from sheep’s or goat’s milk generally contains higher levels of total solids, fat, protein, ash, and salt than its cow’s milk counterpart (27). Organic variants often show reduced levels of saturated fatty acids (SFAs), especially palmitic acid, and increased concentrations of health-promoting fatty acids like oleic acid, conjugated linoleic acid (CLA, specifically rumenic acid), linoleic acid, and alpha-linolenic acid (ALA). These beneficial shifts are especially pronounced when animals are fed diets enriched with grass or olive cake silage. Seasonal changes also influence fatty acid profiles, with springtime milk typically producing Halloumi with a greater proportion of unsaturated fats and fewer saturated on (55). As defined by CYS 94 (1985), authentic Halloumi must be made from sheep’s and/or goat’s milk, with limited inclusion of cow’s milk permitted (53). The process starts by coagulating milk with rennet (0.2–0.4 g/kg), which has minimal impact on proteolytic activity or flavor during ripening (56). Once curds form, they are shaped into rectangular blocks (approximately 10 × 15 × 3 cm), then cooked at temperatures above 90 °C for at least 30 min—an essential step in developing the cheese’s unique texture and thermal stability. Following cooking, the cheese is dry-salted, folded in half, and flavored with crushed mint leaves (*Mentha viridis*). It is then submerged in salted whey, either for short-term storage (about 3 h for fresh consumption) or for longer aging (up to 40 days at 15–20 °C) before packaging as mature Halloumi (53). Additives like buttermilk can influence the cheese’s chemical characteristics by lowering the pH and increasing meltability, oiling-off values, and total volatile fatty acids (TVFA), particularly in Halloumi made from cow or buffalo milk (27). Because it does not melt easily and maintains its firmness when heated, halloumi offers a particular culinary versatility. It is frequently eaten grilled, fried, or shredded in salads, stews, spaghetti, and omelettes (54). The fatty acid composition of the cheese is beneficial to physiological and cardiac health (55). Promising anti-inflammatory and anti-tumor effects can be obtained by including polyunsaturated fatty acids (PUFAs), such as oleic acid and CLA (55, 57).

The native microbial community significantly contributes to Halloumi’s sensory attributes. Despite the high-temperature processing, various microorganisms—including lactic acid bacteria (LAB such as *Lactobacillus*, *Leuconostoc*, *Pediococcus*), halophilic species (*Halomonas*, *Marinilactibacillus*), yeasts, and spore-forming bacteria like *Bacillus*—remain present throughout the production cycle. Notably, *Lactobacillus cypricasei* sp. *nov.*, later reclassified as *Lactobacillus acidipiscis*, was first isolated from Halloumi. Additionally, Halloumi’s microbiota boosts digestive wellness, offers antibacterial qualities, and may prevent spoiling, all of which increase shelf life and improve consumer safety (39). Particularly when stored in the conventional brined whey conditions, its hard mouthfeel and high concentration of salt foil spoiling (54). A common ailment Halloumi suffers from however, is its adulteration by substitution of goat/sheep milk by much cheaper cow’s milk (58). These incidents have warranted greater regulatory scrutiny and establishment of analytical techniques for fraudulent product detection (13).

Whey is generated during manufacture of cheese, casein, and cottage cheese in high volumes. It is the liquid fraction (around 80% of original milk volume taken for coagulation) obtained after curd or coagulated milks solids are separated via acid or enzyme mediated coagulation. It is particularly rich in sulphur-containing and essential amino acids – leucine, isoleucine, and valine to name a few. These amino acids have a direct link with tissue growth and muscle strengthening; hence whey products find themselves wide application as sport and recovery supplements. This by-product is a high value supply of proteins, sugars, fats, minerals, trace elements, vitamins, etc. Whey retains about 36% of energy value of milk from which it is obtained. This protein fraction has found various applications in nutritionally enhancing bakery, beverage, confectionery, and supplement goods. When freshly prepared, the shelf life of whey is 6 h without any processing, as it is kept the active acidity declines and lactic acid and titratable acidity increase (23, 59).

The term *whey protein* encompasses three main forms: whey protein concentrate (WPC), whey protein isolate (WPI), and whey protein hydrolysate (WPH). In production of whey powder, concentration is carried out, wherein salts are removed via membrane technologies such as nanofiltration (60, 61). Whey protein as an inclusion at concentrations lower than 30% can be used to ameliorate the chemical composition of bread without affecting product texture (22). WPC undergoes the least processing and contains between 35 and 80% protein by weight. In contrast, WPI is more extensively refined to eliminate fats and carbohydrates, resulting in a product that is around 90% protein by weight. WPH is treated with enzymes and acids to break down the protein into smaller particles, making it the fastest absorbing of the three types. Protein powders made from whey are rich in amino acids and are usually blends of WPC and WPH (60). WPH exhibits greater thermal stability, emulsifying capacity, foaming properties, digestibility, and absorption, as compared to non-hydrolysed proteins, which can be attributed to presence of bioactive peptides with lower viscosity. In confectionery, WPH can be incorporated in fat-based fillings, improving functional and sensorial attribute of product (62). WPH are widely employed in formulations due to their properties of being antihypertensive, antioxidant, anti-microbial and immune-modulatory (26).

Whey’s surfactant properties make it a good additive for milk based fermented beverage production. It can extend shelf-life by providing bioprotective effect to lactobacillus strains in fermented products (63).

Maillard reaction of whey at 140 °C for 90 min leads to consumption of arginine, histidine, and lactose residues. At the end of this process, notable molecules such as 2-pyrrolecarboxaldehyde, maltol isomer and some antioxidant compounds have been identified. Findings such as these shed light on processing of whey and probable outcomes (64).

Cheese whey is a yellowish-green organic liquid arising cheese production as a by-product. The global estimates of its production approximate at 160 million tonnes per year and depending on milk coagulation it is categorized as sweet (pH 6-7) or acid whey (pH below 5). With suitable processing this can be utilized as substrate in various products (65, 66).

Whey fractions have been processed as additives but can also be stand-alone products such as ricotta cheese and whey-based beverages (61). As per work carried out in (25), an emulsion gel made from pine nut oil, inulin, carrageenan, and WPC (3-4%) formed a stable gel which has excellent moisture and lipid binding properties and can be used as a fat substitute. Table 1 compiles the fermentation techniques and microbial population of traditional dairy emulsions.

**Table 1:** Fermentation technique and microbial population of indigenous dairy emulsions.

Product	Region	Fermentation method	Dominant microbiota/Bioactive compound	Container/Vessel used	Reference
Gariss	Sudan, Somalia	Spontaneous; fed-batch in motion during camel trekking	*Lactobacillus* spp., acetaldehyde, ethanol, organic acids	Skin bag (“Siin”)	(34)
Shubat	Kazakhstan	Controlled fermentation	*Lb. helveticus*, *Lc. lactis*, *Leu. mesenteroides*, *G. candidum*	Bottles, traditional vats	(31, 32, 36, 37, 39)
Chal	Iran	Spontaneous fermentation	*Leuconostoc lactis*, *Lactobacillus plantarum*, *Enterococcus faecium*, antioxidant activity	No specific vessel stated	(33)
Tarag	Mongolia	Boiled then cooled cow/goat milk; cultured with LAB	*Lactobacillus kefiranofaciens*	Wooden vats or clay pots	(18)
Kumys	Central Asia	Ultrafiltration, LAB + yeasts (fermented aerobically)	*Lb. bulgaricus*, *Lb. acidophilus*, *S. lactis*, CO₂ and ethanol	Leather or wooden container	(18)
Rayeb	Egypt, Libya, Tunisia	Gravity-set in earthen pots; no mechanical agitation	*Lactococcus*, *Streptococcus*, *Lactobacillus* spp., diacetyl, acetoin	Earthenware	(48–51)
Laban	Middle East, North Africa	Spontaneous fermentation, sometimes shaken	*Lactococcus lactis*, probiotic peptides, antioxidant activity	Goat leather bag (checoua)	(32, 49, 52)
Dhanaan	Ethiopia	Smoked container, ambient fermentation for 48–72 h	Native microbiota	Smoked plastic container	(12)
Calpis	Japan (inspired by Mongolia)	2-stage fermentation, pasteurized	*Lb. helveticus*, *Saccharomyces cerevisiae*, antihypertensive peptides	Industrial, bottled format	(18)

### 3.5 Other lesser-known indigenous dairy emulsions

Lesser-known dairy emulsions from nomadic and agrarian communities present around the world are a testament of inherent adaptability and resourcefulness of traditional dairying practices. Origins of these emulsion systems are tied to spontaneous fermentation carried out in rudimentary containers or pouches, utilizing natural microflora to produce stable, biologically useful products.

Despite modern disinterest in non-bovine milks, renewed attention is being paid to the functional and nutraceutical properties of these systems, particularly in the context of low-fat diets, protein-rich emulsions, and microbiome diversity (18, 37).

Kumys is a fermented milk emulsion containing both lactic acid and alcohol, traditionally prepared from mare’s milk and is native to Central Asian steppes. Due to its lower fat and casein levels and higher whey protein content, mare’s milk does not coagulate easily with rennet, resulting in the absence of a distinct curd. In modern production, cow’s milk is frequently used as a substitute, with ultrafiltration techniques applied to replicate the protein profile of mare’s milk. The fermentation process typically involves inoculation with a 10% starter culture composed of *Lactobacillus delbrueckii* subsp. *bulgaricus*, *Lb. acidophilus*, and *Saccharomyces lactis*, followed by incubation at 26–28 °C to achieve a firm curd. This is succeeded by aerated fermentation to encourage yeast activity. The final product is mildly alcoholic and carbonated, stored at temperatures at or below 6 °C, and is recognized by its uniform, milky appearance and subtle gray tint. Airag, also known as *tsege* in Mongolia, is a traditional fermentate derived from horse or camel milk. Produced by vigorously agitating milk in leather sacks or wooden containers for one to two days, thereby enhancing oxygen exposure and supporting the lactic acid bacteria (such as *Lactobacillus helveticus* and *Lc. lactis*) alongside native yeast activity, a slight effervescent drink with an ethanol content ranging from 1 to 3% is produced. A type of firm yoghurt produced in Mongolia is Tarag, it is made by fermenting boiled and cooled milk from cows or goats with *Lactobacillus kefiranofaciens*. The result is a dense curd with a pH between 3.6 and 3.9, which also serves as the base for *arkhi*, a distilled alcoholic beverage derived from fermented milk (18).

Edosensuu, is a product resembling soft yoghurt and is a not so commonplace product made from cow’s milk. Fermentation is carried out by *Lactococcus lactis*, *Leuconostoc lactis*, and yeast at ambient temperatures (~17–20 °C), with the final emulsion containing only 0.2% alcohol and 0.8% lactic acid. The last few steps involve skimming of cream after fermentation, leading to a light and slightly acidic emulsion. Drawing inspiration from Mongolian fermented milk beverages, Calpis is a pasteurized dairy emulsion first produced in Japan in 1919. The product undergoes fermentation in two steps: initial processing using cultures of *Lactobacillus helveticus* and *Saccharomyces cerevisiae*, followed by a second step where addition of sugar takes place. Resulting product has sweet and tangy flavour profile which is shelf-stable. The peptides present in this product have been validated for their antihypertensive activity (18).

When carbonation was employed for preservation and enhancement of sensory qualities, particularly in fermented camel milk drinks, utilizing *Lactobacillus delbrueckii* ssp. *bulgaricus* and *Streptococcus thermophilus,* resulting emulsions had lower acidity, reduced proteolysis, and decreased buildup of volatile fatty acids during refrigerated storage (67). This approach is consistent with traditional practices in Central Asia, where yeast-driven carbonation in beverages like kumys and airag also contributes to complex flavors and prolonged shelf life.

Chhurpi produced commonly in Nepal and Bhutan is a hard, energy dense cheese made from yak milk, of great utility especially to herders. Yak milk undergoes long periods of fermentation along with drying to reach to the desired texture.

Buttermilk – conventional or cultured- is a dairy by-product with good emulsifying properties. It is produced from the liquid remnant from churning of milk curd. Further steps involve homogenizing of the curd and dilution with water as necessary. Lassi – salty or sweet- is a fermented beverage made from bovine milk using specific lactic acid bacteria strain. These drinks are easy to digest and can be incorporated with flavours (68).

## 4 Functional and nutritional properties

### 4.1 Natural emulsifiers in traditional dairy (caseins and whey protein concentrates)

In food science, milk proteins, particularly caseins and WPCs, are some of the most researched natural emulsifiers. They can improve texture, stabilize emulsions, and serve as vehicles for transport of bioactive substances thanks to their special physicochemical properties. Caseins, which occur as colloidal micelles in milk, have amphiphilic properties, allowing them to quickly attach to oil–water interfaces and produce thick interfacial coatings that impede droplet coalescence (69). Their flexibility and capacity to create unstructured but solid networks under thermal stress make them very useful in heat-stable emulsions (69).

Whey proteins, which are mostly made up of β-lactoglobulin and α-lactalbumin, additionally exhibit good emulsifying properties, particularly after being denatured by heat or enzyme treatment. Their surface activity gets boosted by these structural changes, which helps them create cohesive interfacial layers with better emulsion qualities (70). Additionally, they are appropriate for a range of food systems, such as beverages, baby formulae, and sports nutrition products, due to their solubility over a broad pH range (70).

The practical utilisation of dairy proteins has been further enhanced by recent developments in their fractionation and processing. Customized protein components with improved emulsification, foaming, and gelation qualities can be produced using methods such ultrafiltration, enzymatic hydrolysis, and spray drying (71). These advancements promote the creation of clean-label and wholesome foods in addition to enhancing both the stability and flavour profile of food products.

### 4.2 Bioactive peptides and antioxidant potential

The proteins have mechanical and biological uses, but they also act as building blocks for a variety of bioactive peptides. Usually inert within the base protein, these peptides are liberated during fermentation, gastrointestinal digestion, or enzymatic hydrolysis. Bioactive peptides identified in fermented dairy emulsions include the tripeptides Val-Pro-Pro and Ile-Pro-Pro, both of which inhibit ACE and contribute to antihypertensive effects. Hydrophobic peptides rich in histidine and tyrosine residues have also been linked with antioxidant capacity. These examples illustrate the molecular mechanisms underlying health-promoting properties (72). Most of these compounds have significant biological functions once they are released, including antibacterial, immune-modulation, antihypertensive, and antioxidant capacities.

Antioxidant peptides thus liberated can bind pro-oxidative metal ions, neutralize free radicals, and prevent the degradation of lipids via peroxidation. Hydrophobic amino acids and particular sequences comprising histidine, proline, tyrosine, and cysteine residues are mainly linked to these actions (72). For instance, because of their enhanced dissolution and surface activity because of hydrolysis, WPCs have been demonstrated to have higher antioxidant activity than intact proteins (73). Their enhanced blending qualities also demonstrate this improvement, making them extremely useful substances in technological and physiological circumstances.

**Figure 2:**
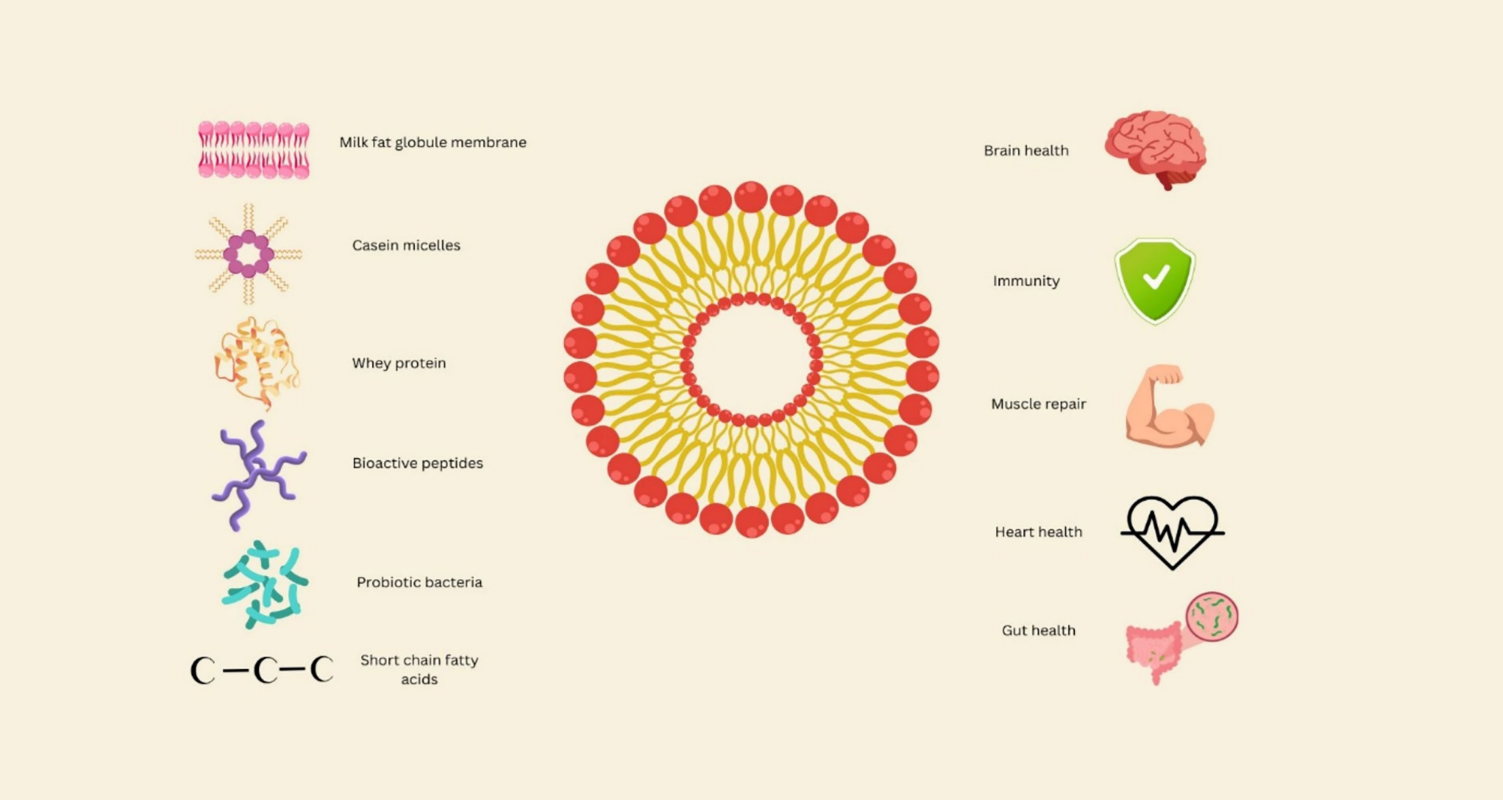
Structural composition and functional bioactivity of traditional dairy emulsions.

Another notable aspect of dairy fermentation techniques is the production of bioactive peptides. Lactic acid bacteria create proteolytic enzymes that hydrolyse milk proteins fractions during fermentation, generating biomolecules having a variety of useful characteristics. Angiotensin converting enzyme (ACE) inhibitory peptides are among them; they work to control blood pressure by preventing angiotensin I from becoming angiotensin II. Bioactive peptides identified in fermented dairy emulsions include the tripeptides Val-Pro-Pro and Ile-Pro-Pro, both of which inhibit ACE and contribute to antihypertensive effects. Hydrophobic peptides rich in histidine and tyrosine residues have also been linked with antioxidant capacity. These examples illustrate the molecular mechanisms underlying health-promoting properties (74). By rupturing bacteria membranes or interfering with metabolic processes, some peptides exhibit antimicrobial action (74). All of these results point to the potential for dairy-derived peptides to influence immunological responses, strengthen antioxidant defences, and improve cardiovascular health. Figure 2 represents the structural composition and functional benefits of traditional dairy emulsions.

### 4.3 Microbiota and fermentation-derived functional benefits

Dairy fermentation has a wider impact on gut microbiota and metabolic wellness in addition to its amino acids release effects. Probiotic and fermentation-derived bioactives can be found in traditional fermented milk goods including yoghurt, kefir, and cultured buttermilk. Fermentation produces metabolites that have prebiotic and antibacterial properties and increases the rate of absorption of nutrients.

Short-chain peptides with immune-stimulating and antibacterial qualities are produced when fermentative microorganisms undergo proteolysis. By encouraging the development of advantageous bacterial communities in the gut and selectively inhibiting harmful bacteria, these beneficial compounds support gastrointestinal equilibrium and structural integrity of barriers (75). Specifically, certain peptides, known as “proteobiotics,” have been found to be signaling compounds that can downregulate virulent genes of an infectious agent modify host immunity (74). These exchanges demonstrate how fermented dairy foods affects host-microbe relationships in an interactive way.

Furthermore, both *in vitro* and *in vivo* investigations have connected peptides with ACE-inhibitory action generated while processing to lower blood pressure and enhanced the function of endothelial cells (74, 75). These results are consistent given the increasing amount of data that suggests using multifunctional fermented goods in dietary plans to control inflammation, oxidative stress, and hypertension.

## 5 Technological approaches for valorization

### 5.1 Nano- and microemulsion techniques

Nano and microemulsion techniques provide a new approach to preserve and valorize traditional dairy emulsions by increasing their solubility, stability, functional profile, and total product shelf life. Nanoemulsions encapsulate heat-sensitive bioactive ingredients, providing high kinetic stability and regulated release inside the dairy matrix. Nanoemulsions have droplet sizes that typically range from 20 to 200 nm. A recent study on bovine colostrum IgG preserved using nanoemulsion found 87% retention following pasteurisation and increased gastrointestinal stability (76). Similar procedures can be used with traditional dairy emulsions such fermented camel milk or peyo, which have immunomodulatory effects but are prone to processing losses.

Furthermore, nanoencapsulating essential oils and extracts from herbs and other plant sources into dairy emulsions has demonstrated promising results. Ultrasound-assisted nanoemulsion techniques were used to incorporate bioactives into the dairy matrix. In a recent study, curcumin-loaded nanoemulsions were introduced into soft cheese, significantly improving its structural integrity, antioxidant potential, and sensory qualities (77). This approach can be used to improve the functional properties of indigenous dairy solids like paneer and khoa, which are currently underutilized despite their emulsifying potential. A study found that adding nanoencapsulated *Heracleum persicum* essential oil (particle size <100 nm) to dairy-based desserts boosted antioxidant activity and microbiological stability during storage (78).

A similar approach can be used to traditional dairy-based desserts such as kulfi and lassi by integrating regional herbs and plant bioactives to create functional dairy products.

Aside from internal formulations, nanoemulsions can be used for preservation and active packaging. A study using clove oil nanoemulsions applied on paneer surface and packaging showed a two-log reduction in microbial load and better sensory quality after 21 days of refrigerated storage (79).

### 5.2 Ultrafiltration for protein recovery

Ultrafiltration is a method that uses pressure to separate protein, lipids, and lactose depending on molecular weight. It is emerging as a sustainable way to valorising traditional dairy streams in order to improve product production, texture, and nutrient retention while also extracting important but underutilized byproducts such as whey, ghee residues, buttermilk, and so on. It is also being used as a sustainable method for recovering and concentrating high-value milk proteins such as whey protein isolates, casein, and immunoglobulins without the use of chemical solvents. A recent study found that ultrafiltration of whole milk at 50 degrees Celsius substantially doubles the permeate flux and achieves 98-99% lactose removal. The resultant concentrate has blander flavour, whiter colour, and higher pH, making it excellent for fermentation or cheese manufacturing (80). A 2023 study examined the concentration of whey recovered from various sources, such as sheep and goats. The researchers discovered that ultrafiltration may recover up to 80% protein from sheep whey and 64% from goat whey. These percentages indicate their suitability for the manufacture of milk protein concentrates (MPC) and whey protein concentrates (WPC). These concentrates can serve as a foundation for traditional dairy products like khoa and rabri (81). Another study used ultrafiltration on sheep and goat whey and found protein contents of up to 7.8% in goat and 16.4% in sheep whey. These were then employed to create a synbiotic kefir with high probiotic viability (82). A similar study on cow whey ultrafiltration was conducted in which UF retentates were added to cheese-like preparations enriched with probiotic cultures, resulting in whey cheese with functional benefits and high acceptability. These products kept the creamy mouthfeel of conventional sweets while improving their nutritional value and shelf stability (83). A similar beneficial kefir drink was created with paneer whey concentrated using ultrafiltration. This ultrafiltrated paneer whey was fortified with kefir culture and fructooligosaccharides, resulting in a protein content of 1.5 g/100 g, an antioxidant activity of 41.3%, and improved shelf stability. This presents a feasible paradigm for value creation and utilisation in Indian dairy systems (84). All of this research indicate that ultrafiltration can be utilized as a toolkit to industrialize, fortify, preserve, and improve the nutritional and sensory properties of traditional dairy emulsions.

### 5.3 Spray drying of dairy emulsion by-products

Spray drying is the process of atomising a liquid field using quick heat drying and then collecting the powder, so transforming perishable liquid items into shelf stable powders. This procedure is ideal for making and stabilising whey, buttermilk, or skim milk powders for both conventional and modern applications. It is developing as an efficient technique for reducing waste and increasing the market viability of traditional dairy systems by conserving functional proteins, prolonging shelf life, and allowing for the flexible usage of dried traditional foods such as lassi and khoa (85) used a pulse spray drying technique to create skim milk powder with over 98% protein retention and fine particle size (<10 μm), allowing for quick rehydration and minimal moisture and bulk density changes during storage, making it ideal for traditional beverages like lassis. A comparative proteomic study conducted by Zhou et al. on spray and freeze dried milk powders from bovine, goat, and horse sources found that spray dried versions had smaller particle size but low solubility and immune protein content, implying that optimisation of specific spray profiles is required for optimal retention (86). Similarly, another study found 61% solid recovery in camel milk whey with low protein loss, which is required to preserve the nutritional and medicinal qualities of camel milk (87). Another study on large-scale whey protein concentrate manufacturing revealed that spray drying maintains bioactive proteins under standard pasteurisation settings to the same extent as freeze drying with minimum Millard response (88). As a result, we can infer that, with adequate optimisation, spray drying can be an effective technology for traditional dairy utilisation and product innovation. Table 2 compiles the techniques utilized for valorization of dairy emulsions.

**Table 2:** Techniques for valorization of dairy emulsions.

Dairy matrix /Additive	Process	Valorization technique	Functional outcome	Study (Year)
Skimmed bovine milk	Pulse spray drying	Retentate powder preservation	Visibly fine particles; instant solubility	(85)
Soft cheese with curcumin NE	Ultrasonication nanoemulsion	Curcumin nanoemulsion	Increased Shelf life, antioxidant, antimicrobial activity, increased Sensory quality by 150%; better microstructure	(77)
Milk cream	Microfluidization, powder by spray drying	Nano-curcumin encapsulation in cream powder	93% EE; 88% Bioaccessibility	(68)
Camel whey	spray drying	Low-temp antioxidant protection	61% solids yield; preserved immunoglobulins	(87)
Sour buttermilk	Reverse osmosis pre concentration, spray drying	Fermented-source powder valorization	54% protein; high antioxidant content	(132)
Whey protein concentrate	Spray drying, freeze drying	WPC functional recovery	Antioxidant bioactivity preserved	(88)
Sheep/goat whey	Ultrafiltration + probiotic addition	Protein and culture retention	High-protein fermented whey desserts	(82)
Whole milk	Ultrafiltration	Milk protein fortification	Reduced lactose; increased protein content	(133)
Camel milk	Emulsion formulation	Camel dairy valorization	Function potential mapping	(134)

### 5.4 Incorporation into functional and infant food formulations

Given their distinct compositional and colloidal features, indigenous dairy emulsions can be used to boost the natural bioactivity of functional foods and infant food formulations. Several fortified formulations have been created employing milk fat globule membranes (MFGM), whey protein concentrates (WPC), and natural polysaccharides, merging traditional dairy systems with modern nutritional needs. Kondrashina et al. (89) enriched newborn formulae with 38% dairy cream to increase the amount of MFGM, which is known to have immunomodulatory and cognitive development benefits. The study discovered that high cream formula generated a greater spectrum of bioactive peptides during enhanced gastric digestion and supported anti-inflammatory responses and intestinal recovery similar to human milk (89). Another study examined two formulas: one with whey-derived extracellular vesicles (WPC-EV) and another with phospholipids (WPC-PL). These dairy emulsified formulations increased hippocampus myelination, lipolysis kinetics, and altered plasma lipidomics, demonstrating MFGM’s significance in neurodevelopment (90). Because of its prebiotic, hypoallergenic, and improved digestion features, hydrolysed whey protein is increasingly being used in newborn diets. A study looked at many models for whey protein concentrates (WPC) in infant feeding formulae. They discovered that adding WPC improved powder solubility, decreased stomach coagulation, and increased peptide-mediated antioxidant activity (91). Another study introduced a low-energy nanoemulsion system containing fish oil and gamma oryzanol into stirred yoghurt. Dairy proteins and phospholipids stabilized this nanoemulsion, resulting in droplets with a size of less than 200 nm and remarkable oxidative stability. The yoghurt kept over 95% of its EPA and DHA content while having much reduced peroxide readings. These methodologies can be used to traditional dairy emulsions such as lassi, matta, and fermented camel milk, where lipid distribution in low viscosity is desirable (92). Table 3 describes various dairy emulsion derived functional and infant foods.

**Table 3:** Dairy emulsion derived functional and infant foo.

Product	Emulsion	Processing method	Valorization technique	Benefits	Reference
Preterm infant formula	Phospholipids/WPC derived extracellular vesicles	Ultracentrifugation, homogenization	Replacing synthetic emulsifiers with dairy derived lipid carriers	Improved hippocampal myelination; enhanced lypolysis	(90)
Stirred yogurt	Inulin–WPC microcapsules	Electrospraying (1–5 μm)	Protein–prebiotic encapsulation of probiotics	robust gastric survival, >90% probiotic viability	(135)
LC-IMF/HC-IMF infant formulas	Dairy cream–enriched MFGM	High-pressure homogenization + microfluidization	Emulsion fortification with natural MFGM	gut barrier support, Broad spectrum of bioactive peptides	(89)
Model infant formula	Whey protein hydrolysate (WPH)	Controlled spray drying with enzymatic pre-treatment	Protein hydrolysis for hypoallergenic formulation	Improved solubility; reduced gastric coagulation	(91)
Fish oil–fortified yogurt	Fish oil/γ-oryzanol nanoemulsion	Low-energy emulsification, dairy matrix integration	Nanoemulsion of lipophilic bioactives into fermented emulsion	reduced syneresis, >95% EPA/DHA retention	(86)

## 6 Challenges and sustainability perspectives

### 6.1 Industrialization vs. preservation of traditional methods

The rapid industrialization of fermented dairy production has led to increased availability, standardized quality, and longer shelf life of dairy products. However, these benefits have come at the cost of eroding traditional knowledge systems and microbial biodiversity intrinsic to artisanal fermentations (93). Traditional fermented dairy products, such as dahi, mattha, lassi, and camel milk-derived suusac, are often produced using spontaneous fermentation methods relying on indigenous microbiota and community-specific processing techniques. Industrial systems, on the other hand, prioritize pasteurization, monoculture starter cultures, and automation to ensure microbial consistency and safety (94). While safety and scalability are legitimate concerns, the imposition of rigid industrial standards has marginalized traditional producers and homogenized flavor profiles (95). Moreover, standardization has led to the loss of regional microbial strains with unique probiotic and bioactive potential. For instance, several studies on Himalayan and north-eastern Indian fermented dairy products have shown the presence of novel strains of Lactobacillus and Leuconostoc with promising therapeutic properties (96).

**Figure 3:**
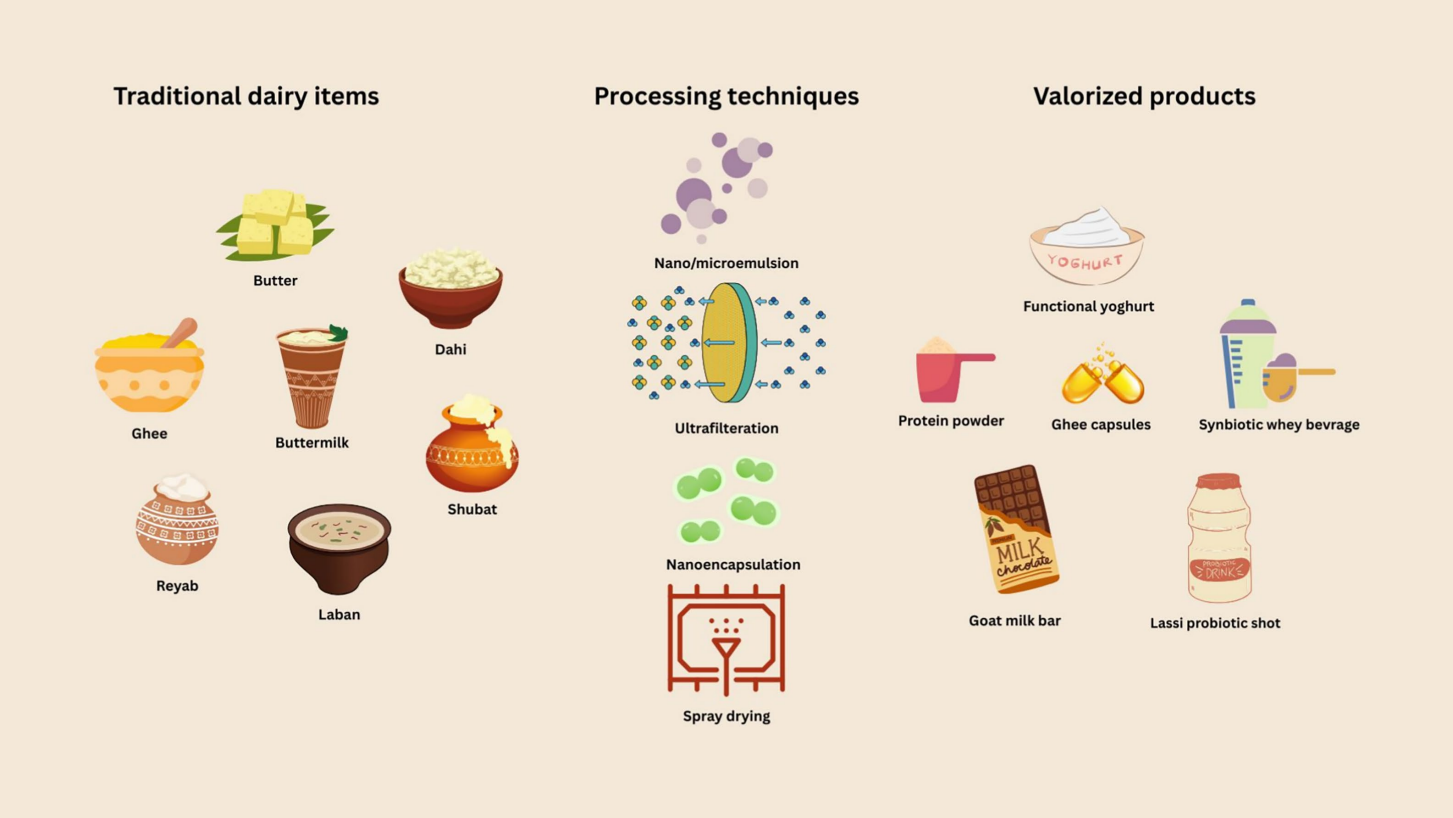
Technological valorization pathways of indigenous dairy emulsions.

Preserving traditional fermentation methods necessitates an integrative model where modern microbiological techniques validate indigenous practices without displacing them. Microbial banking, participatory fermentation mapping, and the development of hybrid starter cultures that incorporate traditional strains are promising strategies. Hybrid strategies represent a promising path forward. These include the development of industrial starter cultures derived from indigenous microbial strains, participatory microbial mapping of traditional products, and collaborative innovation between local producers and food scientists. Such approaches preserve authenticity while enhancing safety, scalability, and sustainability (97, 98). In this context, the valorization of traditional products through Geographical Indication (GI) tagging and ethical sourcing frameworks also contributes to the preservation of food heritage and rural economies. Figure 3 describes the various technological pathways for valorization of traditional dairy products.

### 6.2 Regulatory and safety considerations

One of the core tensions in scaling traditional dairy fermentation lies in navigating the complex regulatory landscape. In India, for example, the Food Safety and Standards Authority of India (FSSAI) provides guidelines that often do not adequately address the microbial diversity or variability inherent in traditional fermented foods (99). Similar regulatory rigidity is seen in Codex Alimentarius and EU frameworks, which tend to privilege industrial models with quantifiable microbial loads and traceability metric (100).

Traditional fermented dairy products are frequently produced in informal settings with limited access to microbiological testing or pathogen control mechanisms. Despite this numerous studies have shown that artisanal fermented dairy contains high levels of lactic acid bacteria (LAB) with strong antimicrobial properties, capable of suppressing pathogenic bacteria such as *Listeria monocytogenes* and *Escherichia coli* (101, 102). Thus, blanket regulations that disqualify such products on the basis of non-standardized practices ignore their inherent safety mechanisms.

A more inclusive regulatory framework should accommodate risk-based evaluations that consider traditional fermentation environments and integrate safety training for artisanal producers (103). Moreover, mobile testing kits, community-led microbial monitoring, and institutional support for upgrading infrastructure can bridge the gap between traditional authenticity and modern safety standards (104).

### 6.3 Supply chain, storage, and scalability barriers

Supply chain limitations present another significant barrier to the sustainability of traditional fermented dairy systems. These products are highly perishable, requiring immediate consumption or cold chain logistics that are often lacking in rural or arid regions (93). Seasonal milk production, especially in nomadic or pastoralist communities, leads to fluctuations in availability and difficulty in maintaining steady market supply (105).

Furthermore, the packaging and storage practices for these products often do not conform to the standards required for modern retail environments. Traditional storage in earthen pots or animal-skin containers, though effective at the community level, poses challenges for urban distribution (106). Innovations such as solar-powered refrigeration, biodegradable antimicrobial packaging, and lyophilization (freeze-drying) of starter cultures offer scalable solutions (107). Additionally, cooperative-based marketing and supply chain clustering, exemplified by AMUL and other Indian dairy federations, demonstrate the potential of collective bargaining and resource pooling to overcome distributional constraints (108). Aligning traditional practices with modern supply chain logistics requires not replacement but adaptation a sustainability model that respects both heritage and functionality.

## 7 Contribution to climate resilience and food security

### 7.1 Resilient livestock and arid-zone dairy systems

Traditional fermented dairy products are closely interwoven with the livestock systems of arid and semi-arid regions. Animals such as camels, goats, sheep, and indigenous cattle breeds (e.g., Gir, Sahiwal, Tharparkar) are naturally adapted to thrive under extreme temperatures, water scarcity, and poor grazing conditions (106, 109). These livestock provide highly nutritious milk that is often fermented into products like suusac (fermented camel milk), goat milk dahi, or sheep milk laban. Unlike high-yield exotic cattle breeds, these indigenous animals are disease-resistant and well-suited for extensive pastoral systems that are increasingly under threat from climate variability. Camelids, in particular, have been called “livestock of the future” due to their ability to survive in harsh deserts while producing milk rich in lactoferrin, lysozyme, and immunoglobulins components with both nutritional and antimicrobial properties (110, 111). The fermentation of milk in arid zones is not just a preservation technique but a climate-resilience strategy. It extends milk’s shelf-life without refrigeration, reduces post-harvest losses, and ensures year-round availability of dairy nutrition (93).

Furthermore, the spontaneous fermentation used in these systems often leverages robust microbial consortia that are tolerant to heat and pH stress making them ecologically stable and self-renewing (112). Supporting these systems involves policies that protect grazing rights, mobile veterinary services for pastoralists, and research into heat-tolerant probiotic strains derived from desert microbial communities (113). These actions enhance the contribution of fermented dairy to both local food security and global biodiversity conservation.

### 7.2 Role in circular bioeconomy and zero-waste strategies

**Figure 4:**
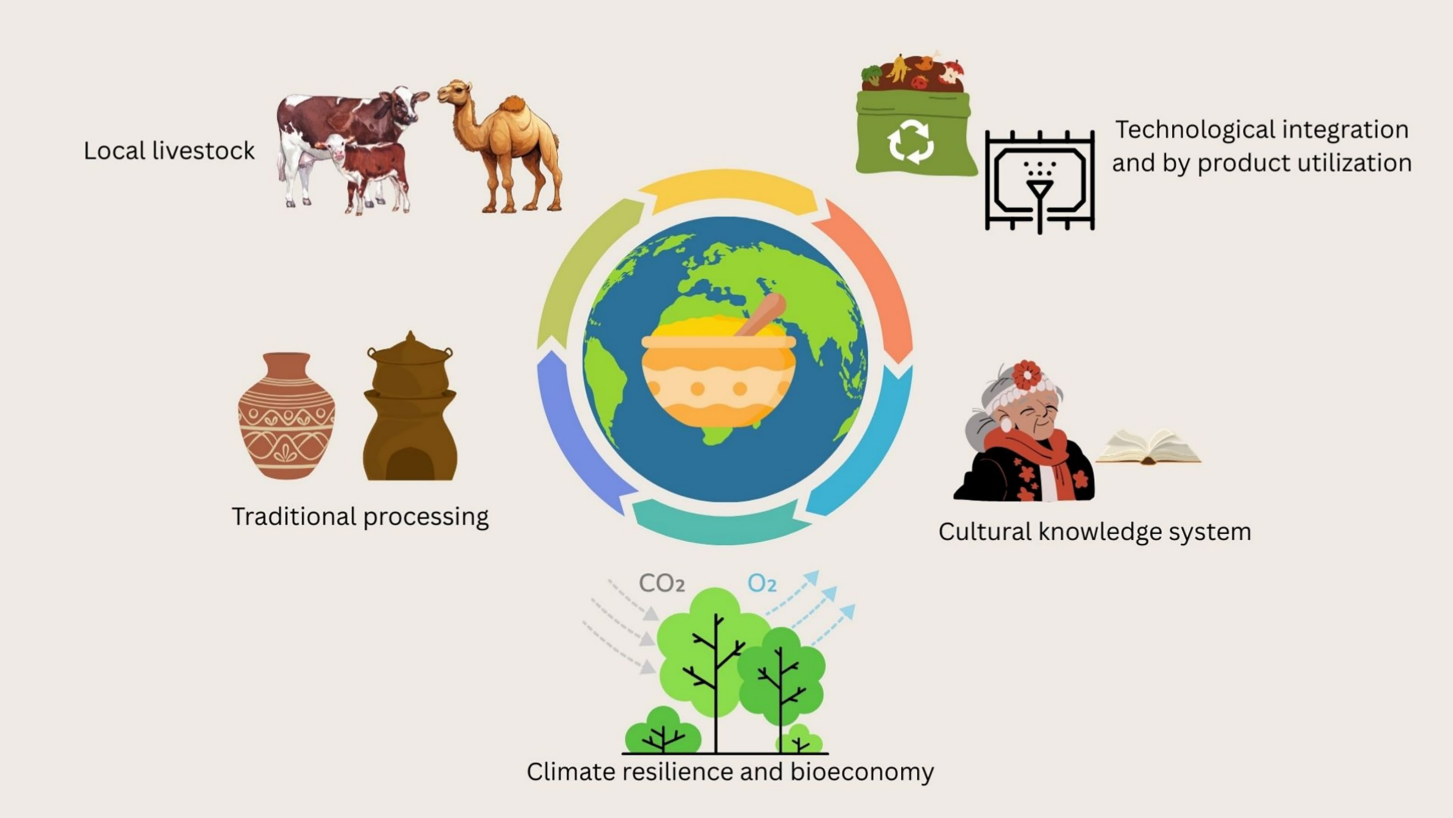
Traditional dairy emulsions in circular and resilient food systems.

Traditional dairy fermentation practices embody many principles of the circular bioeconomy. Milk that would otherwise spoil due to lack of refrigeration is fermented into products that not only last longer but provide enhanced health benefits. Similarly, by-products such as whey, ghee residue, and buttermilk are routinely repurposed into animal feed, probiotic drinks, or substrates for secondary fermentation (114, 115). Whey, for example, contains lactose, proteins, and micronutrients that can be used for the production of ethanol, bioplastics, and single-cell proteins (116). In India, the use of chhaach (spiced buttermilk) and mattha (whey-based drinks) showcases zero-waste fermentation practices rooted in culinary traditions. In addition, innovations like anaerobic digestion of dairy waste into biogas or its conversion into bio-fertilizers for local agriculture are being adopted at pilot scales in community dairies (117). Another aspect of sustainability is packaging. Traditional packaging, such as banana leaves or clay pots, is biodegradable, although unsuitable for modern retail. Research is now focused on designing modern packaging using biopolymers or whey-protein films with antimicrobial properties (107). Such eco-friendly packaging extends shelf life while minimizing plastic use, thus closing resource loops in the dairy supply chain. Integrating traditional dairy fermentation into the formal circular bioeconomy requires aligning community practices with green technologies, ensuring that smallholder knowledge systems are not marginalized but empowered in the process. Figure 4 depicts traditional dairy emulsions in circular & resilient food systems.

### 7.3 Socioeconomic impact on local and nomadic communities

Fermented dairy products serve as more than just nutrition sources in rural and nomadic societies they are embedded in livelihood strategies, gender roles, and cultural identity. In Rajasthan, Gujarat, Maharashtra, and parts of Central Asia, women are the custodians of dairy fermentation, preserving strains, regulating temperature and pH through experience, and passing knowledge intergenerationally (118). This feminization of traditional dairy has both nutritional and economic dimensions. Women- led self-help groups and Farmer Producer Organizations (FPOs) have demonstrated the potential to commercialize traditional products like lassi, chhurpi, and ghee with minimal capital investment (119). As demand for natural, artisanal, and probiotic-rich products grows in urban markets, these traditional fermentations offer pathways for rural value addition and women’s empowerment. Moreover, for nomadic communities such as the Raika in India, the Tuareg in North Africa, or the Bedouins in the Middle East, fermented milk serves as a staple during transhumance (seasonal migration), providing stable calories, hydration, and gut health under uncertain food conditions. Policies that support mobile dairy units, community refrigeration systems, and cultural labeling (like GI tags or “heritage food” certifications) can safeguard both the product and the producer. From a food security perspective, fermented dairy is a strategic resource in times of drought, conflict, or migration events that are becoming more frequent under climate change scenarios (99). Incorporating these traditional systems into national nutrition and disaster-resilience plans can provide decentralized, culturally familiar, and nutritionally dense food options to vulnerable populations.

## 8 Future directions and research needs

### 8.1 Molecular characterization and metabolomics

Advancements in systems biology have transformed our understanding of fermented dairy products, especially those produced through traditional methods. Techniques such as metagenomics, transcriptomics, proteomics, and metabolomics now enable the in-depth characterization of microbial ecosystems and bioactive compounds present in artisanal dairy fermentations (120). Traditional fermented milk products are typically produced via spontaneous fermentation, where undefined and diverse microbial consortia drive lactic acid production, flavor development, and probiotic activity. Recent studies have revealed that many of these microbial communities consist of novel strains of Lactobacillus, Leuconostoc, Weissella, and Pediococcus that are not found in industrial cultures (96, 121). These indigenous strains often show tolerance to high temperatures, low water activity, and antimicrobial resistance, making them ideal for use in climate-resilient fermentation systems (122).

Metabolomic profiling further allows the identification of short-chain fatty acids (SCFAs), bacteriocins, exopolysaccharides, vitamins (such as B12 and folate), and bioactive peptides that confer functional health benefits (123). For example, camel milk fermentation produces bioactive peptides with antihypertensive and antidiabetic properties (124). However, these health claims need to be validated via clinical and preclinical models under Good Laboratory Practice (GLP) protocols.

Future research should focus on constructing strain libraries from underexplored regions (e.g., Central India, Northeast Himalayas, Sahel, Horn of Africa), sequencing their genomes, and assessing synergistic microbial interactions. Equally important is the creation of open-access databases cataloging traditional dairy microbes along with their biochemical traits and health-promoting properties (125). This integration will bridge traditional knowledge systems with contemporary biotechnological innovation.

### 8.2 Packaging, shelf-life, and market integration

As consumer demand grows for probiotic-rich, artisanal dairy products, enhancing shelf-life and retail readiness becomes critical. Many traditional fermented products have limited commercial viability due to short shelf life, perishability, and variable sensory profiles, particularly in the absence of preservatives and refrigeration (126). Innovative packaging technologies offer potential solutions. For instance, antimicrobial films made from whey protein, chitosan, or plant polyphenols have shown effectiveness in inhibiting spoilage microbes while maintaining probiotic viability (127). Modified atmosphere packaging (MAP), which involves replacing oxygen with inert gaseslike nitrogen or carbon dioxide, can also reduce oxidative degradation and microbial spoilage in fermented dairy (128). Freeze-drying (lyophilization) is another area of interest, enabling the long-term preservation of starter cultures or even finished fermented products without compromising microbial viability. Lyophilized camel milk kefir and goat dahi have shown promising shelf stability and reconstitution ability (129). Market integration also requires robust branding, storytelling, and compliance with food safety certifications such as FSSAI (India), USDA Organic, or EU Protected Designation of Origin (PDO). Training programs for rural entrepreneurs on food safety, digital marketing, and sustainable packaging can open new markets while ensuring product integrity. Farmer Producer Organizations (FPOs) and dairy collectives can act as incubators for scale-up, offering decentralized pasteurization, packaging, and distribution infrastructure (119).

Future research must address sensory stabilization, standardization of fermentation parameters, and probiotic dose consistency. Collaborations between food technologists, microbiologists, and ethnographers will ensure that commercialization does not come at the cost of authenticity.

### 8.3 Role in personalized and preventive nutrition

With the growing interest in the human microbiome, traditional fermented dairy is being re-evaluated as a source of personalized nutrition and preventive medicine. Variability in individual gut microbiota means that different people may respond differently to the same probiotic strains or fermented foods (56, 130). This opens opportunities to develop microbiome-based dietary interventions, particularly in populations with high prevalence of metabolic disorders, gastrointestinal diseases, or nutritional deficiencies. Camel milk, for example, has shown promise in managing Type 1 diabetes due to its insulin-like proteins, while goat milk dahi has been found to reduce inflammatory markers in irritable bowel syndrome (IBS) models (131). However, large-scale clinical trials remain sparse and are a key area for future inquiry. Additionally, traditional fermentation techniques can be leveraged to tailor dairy products for specific demographic needs such as high-calcium dahi for postmenopausal women, vitamin B12 enriched kefir for vegans, or iron-fortified mattha for adolescent girls in anemia-prone regions (9, 95).

Nutrigenomics and metabolomics-based personalization of traditional fermented foods can improve compliance, cultural acceptance, and health outcomes. However, regulatory frameworks must evolve to allow structure–function claims backed by scientific evidence. Interdisciplinary platforms involving public health professionals, traditional knowledge holders, and biotech innovators will be essential for building evidence-based, culturally rooted interventions in preventive nutrition.

## 9 Limitations

Despite the extensive body of literature on traditional dairy emulsions, several limitations must be acknowledged. First, there are gaps in the literature, particularly in relation to comprehensive studies on the biochemical composition, functional properties, and long-term health benefits of these products. While many studies focus on specific emulsions, few offer a holistic view of their role in modern diets or their broader environmental impact. Second, the heterogeneity of traditional practices across different regions makes it challenging to generalize findings. Variations in milk sources, production methods, and cultural contexts can result in significant differences in product characteristics and health outcomes, limiting the applicability of results to global populations. Third, while there are numerous health claims associated with traditional dairy emulsions, there is a lack of clinical evidence to support many of these claims. Most studies are observational or rely on *in vitro* models, and there is a need for rigorous clinical trials to establish clear causal relationships between consumption of these emulsions and specific health benefits. Finally, publication bias is a potential concern, as studies with positive outcomes are more likely to be published, while those with negative or inconclusive results may remain unpublished, skewing the overall understanding of the efficacy of traditional dairy emulsions in promoting health.

## 10 Conclusion

This review explore the diverse terrain of traditional dairy emulsions that are nutrient-dense, culturally anchored, and technologically relevant. These dairy emulsions make important contributions to edible biodiversity, rural livelihoods, and food heritage through local ecology and generational practices. From the use of ghee in the rituals and medicine to the fermentation of camel milk in desert locations and probiotic laban and reyab, these goods demonstrate a strong intertwining of resilience, cuisine, and identity. Traditional dairy products have complex oil in water systems such as casein micelles, whey protein, and milk fat globule membranes, which distinguishes their nutritional profile. When carefully incorporated into infant and functional foods, they provide a variety of antioxidant chemicals, bioactive peptides, and living microbes that promote metabolic balance, intestinal health, and neurodevelopment. Heat-sensitive compounds can be preserved using specialized valorisation processes such as nano and microemulsion techniques, while ultrafiltration can be used to concentrate and strengthen these products. Other processes, like as spray drying, can be used to transform typical dairy wastes into shelf stable powders, while encapsulation can be utilized to give probiotics and lipophilic nutrients. By maintaining the art of traditional dairy product making, we can assure equitable access to processing technologies and develop supportive regulatory frameworks that support circular bioeconomy models. By combining modern food science with traditional wisdom, we may develop novel traditional dairy-based dishes that reflect cultural heritage while improving nutritional value and also by leveraging modern technologies such as nanoemulsions and ultrafiltration, these products can be valorized to enhance their health benefits while preserving their cultural identity. Integrating traditional knowledge with scientific advancements offers a pathway to enhancing global nutrition, especially in regions relying on indigenous dairy systems.
